# Commentary: mechanistic considerations for associations between formaldehyde exposure and nasopharyngeal carcinoma

**DOI:** 10.1186/1476-069X-8-53

**Published:** 2009-11-25

**Authors:** Chad M Thompson, Roland C Grafström

**Affiliations:** 1 ToxStrategies, Inc, 23501 Cinco Ranch Blvd, Suite G265, Katy, TX 77494, USA; 2 Institute of Environmental Medicine, Karolinska Institutet, SE-171 77 Stockholm, Sweden; 3 VTT Technical Research Centre of Finland, Medical Biotechnology, PO Box 106, FI-20521 Turku, Finland

## Abstract

Occupational exposure to formaldehyde has been linked to nasopharyngeal carcinoma. To date, mechanistic explanations for this association have primarily focused on formaldehyde-induced cytotoxicity, regenerative hyperplasia and DNA damage. However, recent studies broaden the potential mechanisms as it is now well established that formaldehyde dehydrogenase, identical to *S*-nitrosoglutathione reductase, is an important mediator of cGMP-independent nitric oxide signaling pathways. We have previously described mechanisms by which formaldehyde can influence nitrosothiol homeostasis thereby leading to changes in pulmonary physiology. Considering evidences that nitrosothiols govern the Epstein-Barr virus infection cycle, and that the virus is strongly implicated in the etiology of nasopharyngeal carcinoma, studies are needed to examine the potential for formaldehyde to reactivate the Epstein-Barr virus as well as additively or synergistically interact with the virus to potentiate epithelial cell transformation.

## Introduction

The International Agency for Research on Cancer (IARC) has classified formaldehyde as a human carcinogen based, in part, on epidemiological evidence that formaldehyde increases the risk of nasopharyngeal carcinoma (NPC) [1-4]. This cancer exhibits remarkable geographical distribution that is posited to result from environmental factors, host genetic factors (i.e. race), and genetic variation in the gammaherpesvirus Epstein-Barr virus (EBV) that is widely believed to play a role in the etiology of NPC [5-9]. Mechanisms posited to explain formaldehyde carcinogenicity have invoked evidence for cytotoxicity, compensatory cell proliferation, and genotoxicity in animal bioassays [4,10-13]. However, mathematical models of the human respiratory passages indicate that the highest doses of formaldehyde are predicted to be above the hard palate, with relatively less vapor reaching the nasopharynx [14]. Histopathological samples from workers exposed to formaldehyde exhibit only mild nasal tissue pathology [4,15], suggesting that tissue damage might not occur in more distal regions. Thus, available data do not paint a coherent mechanism for formaldehyde to increase the risk of NPC, and moreover the association continues to be disputed [16-18].

**Figure 1:**
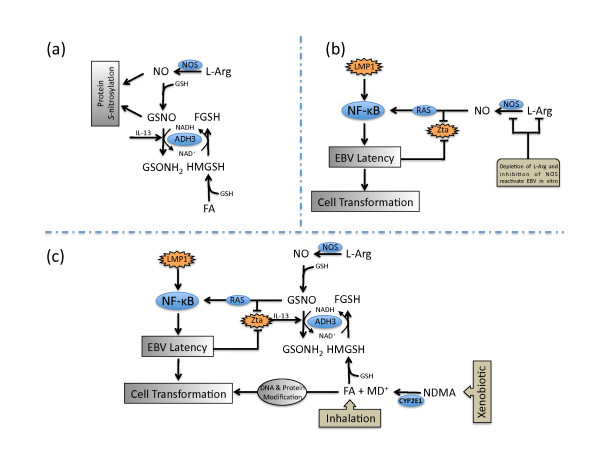
**Simplified hypothetical model for formaldehyde interaction with EBV**. a) Alcohol dehydrogenase 3 (ADH3) catalyzes the oxidation of GSH-conjugated formaldehyde (*S*-hydroxymethylglutathione, HMGSH) to *S*-formylglutathione (FGSH), and the reduction of GSH-conjugated NO (*S*-nitrosoglutathione, GSNO) to glutathione sulfinamide (GSONH_2_). Both NO and GSNO regulate protein function by *S*-nitrosylating cysteine residues, albeit with differential specificity. Excessive formaldehyde can accelerate GSNO reduction via NAD^+^/NADH cofactor recycling leading to decreased cellular nitrosothiols. Interleukin-13 has recently been shown to increase ADH3 expression. b) EBV latency programs that contain LMP1 promote latency, in part, through activation of NF-κB pathways. NO appears to be required for latency in programs that lack LMP1, as NOS inhibition can reactivate the EBV in vitro. Similar to LMP1, this process likely involves activation of NF-κB. This NO-mediated regulation of EBV is cGMP-independent and involves *S*-nitrosylation of RAS by NO and/or GSNO. In addition, binding of the early lytic transcription factor, Zta, to DNA is inhibited by redox and nitrosative modification of cysteine residues. c) Nitrosothiols influence the EBV life cycle. Increased ADH3 expression or excessive formaldehyde exposure from xenobiotic metabolism (e.g. *N*-nitrosodimethylamine, NDMA) or exogenous sources may accelerate GSNO reduction thereby promoting EBV reactivation. Zta may also increase IL-13, which may in turn increase ADH3 expression. In addition to EBV reactivation, formaldehyde mediated protein modification and DNA reactivity can interact with established EBV latency programs to promote epithelial cell transformation.

Few articles published on formaldehyde toxicity over the past decade have acknowledged that the class III alcohol dehydrogenase (ADH3), also termed formaldehyde dehydrogenase and *S*-nitrosoglutathione (GSNO) reductase, is a key mediator of the nitric oxide (NO)-related post-translational protein modification known as *S*-nitrosylation (Figure 1a) [19-24]. The *S*-nitrosylation of protein cysteine residues by NO and GSNO regulates a broad spectrum of cellular proteins and functions [25-36]. Dysregulation of nitrosothiol homeostasis has been implicated in diseases of the central nervous system, cardiovascular system, and lung [37-43]. We have previously described how ADH3 and nitrosothiols influence pulmonary physiology, and how formaldehyde might influence nitrosothiol homeostasis [24,44,45]. Considering that nitrosothiols are implicated in the regulation of EBV and other viruses [28,29,32,46], herein we describe mechanisms by which formaldehyde might influence the reactivation of the EBV and additionally interact with EBV latency programs to potentiate epithelial cell transformation.

## Discussion

### (a) The EBV Life Cycle and NPC

The World Health Organization has periodically classified NPC based on histology, yet all NPC appears to be variants of squamous cell carcinoma [5]. Though NPC risk varies geographically, nearly all cases and types of NPC demonstrate EBV-positive cells and thus EBV is strongly implicated in the etiology of NPC [5,7,8]. Over 90% of the population is EBV positive, yet EBV is rarely detected in normal human epithelial biopsies, and when it is detected, it is almost always in carcinomas and in the latent phase [6,8,47]. The EBV exhibits three latency programs [48]. Latency III is the least restrictive and involves the expression of six nuclear antigens (EBNA1-6), three latent membrane proteins (LMP1, LMP2A, and LMP2B), and untranslated nuclear RNAs. Latency I is the most restrictive and expresses LMP2A and EBNA1 and is associated with memory B-cells. The Latency II program expresses EBNA1 and the three LMPs, and is the program associated with NPC [48]. These latency programs and other factors compete to repress and promote the activity of the EBV lytic transcription factor Zta [47]. Importantly, both EBV reactivation and EBV latency programs are thought to contribute to the etiology of NPC [6,8,49-52]. The former process may increase the risk of infection to target epithelial cells, whereas the latter may increase the risk of cell transformation. The potential influence of formaldehyde on these processes is described in the sections that follow.

### (b) Nitrosothiols Influence the EBV Life Cycle

In 1968, the EBV was reported to be reactivated in cells cultured in media deficient in the nonessential amino acid and NO precursor L-arginine [53]. Over 25 years later, NO synthase (NOS) inhibitors were shown to reactivate EBV in human lymphocytes exhibiting Type I latency but not Type III [29]. This specificity was posited to relate to the fact that LMP1 (not present in Type I latency) and NO converge on similar signaling pathways such as NF-κB [29,34]. NO also prevented B-cell apoptosis, which is consistent with evidence that LMP1 promotes uncontrolled B-cell proliferation [29,48]. This suppression of the lytic phase and apoptosis by NO was reportedly mediated through cGMP-independent signaling pathways, and likely involves nitrosothiol modification of p21 Ras [29,34,54]. NO also suppresses the lytic phase of EBV in epithelial cell lines; and moreover, the established induction of EBV reactivation by TPA (12-*O*-tetradecanoylphorbol-13 acetate) coincided with decreased levels of NOS [55]. It is also noteworthy that NO inhibition increased the expression of the early lytic transcription factor Zta in both lymphocytes and epithelial cells [29,55]. The binding of this viral transcription factor to DNA is reported to be inhibited by both redox and nitrosative mechanisms that involve critical cysteine residues [28]. Interestingly, Zta is reported to induce the expression of interleukin-13, which may in turn up-regulate ADH3 [56,57]. Although further studies are needed to examine these responses together in individual cell types and in in vivo systems, the data suggest that nitrosothiol homeostasis plays a dual role in promoting the latent phase and inhibiting the lytic phase of the EBV in lymphocytes and epithelial cells expressing certain latency programs (Figure 1b).

To date, little is known about what conditions might lead to altered nitrosothiol homeostasis in vivo. It is becoming increasing clear, however, that environmental exposures to allergens can elevate ADH3 expression in airways thereby decreasing nitrosothiol levels sufficiently to cause patent changes in pulmonary physiology [41,57,58]. Moreover, evidence suggests that exposure to relatively high levels of formaldehyde vapor can lead to immune responses, elevated ADH3, and increased GSNO breakdown in rodent airways [45,59,60]. Additionally, formaldehyde can accelerate GSNO reduction several fold in cell and cell-free systems through NAD^+^/NADH cofactor recycling on ADH3, as well as decrease cellular nitrosothiol levels in vitro (Figure 1a)) [61,62]. Together, these data suggest that formaldehyde can accelerate GSNO reduction, which might promote EBV reactivation in cells lining the airways (Figure 1c). This reactivation, in turn, could lead to increased viral shedding in nasopharyngeal lymphocytes and subsequent infection of basal epithelial cells [8]. It is notable, though circumstantial, that the relative risk of NPC among occupationally exposed workers is reported to be stronger among EBV seropositive individuals [2].

### (c) EBV Latency and Cell Transformation

Several mechanisms are implicated in EBV-mediated cell transformation. EBNA1, EBNA2, and LMP1 have all been shown to induce oxidative stress, genomic instability, or DNA repair inhibition [63-66]. In fact, LMP1 is posited to be an important oncogene and a key modulator in the pathogenesis of NPC through activation of NF-κB signaling pathways related to cell cycle control, apoptosis and transformation [51]. LMP1 acts as a ligand-independent CD40 receptor that functions, in part, by preventing the ubiquitination of TNF-receptor associated factors [50]. Other EBV proteins manipulate the fate of host proteins by accelerating or decelerating their degradation through the ubiquitin-proteosome system, while simultaneously decelerating their own ubiquitination and degradation [50,67]. These myriad effects imparted by EBV latency programs likely perturb many cellular functions in subtle ways that can increase spontaneous transformation or otherwise potentiate cells for transformation. As noted previously, the etiology of NPC involves host, viral and environmental factors. Considering that the functions of many proteins may be modulated by *S*-nitrosylation, it is conceivable that excess formaldehyde exposure may further alter protein functions in EBV-positive cells. Additionally, formaldehyde itself can deplete GSH levels and can react with proteins and peptides [68-70]. Recent genomics analyses in yeast cells clearly indicate that oxidative stress pathways are induced by formaldehyde exposure [70]. These analyses also identified protein fate (processing and degradation, folding and stabilization, and ubiquitination pathways) as a major category of altered gene expression; formaldehyde induced 14-19% of genes involved in ubiquitination processes, whereas methanol (which is oxidized to formaldehyde) induced only 1% of these genes [70]. While this simple eukaryotic system may not directly inform in vivo mammalian tissue responses, there is little reason to believe that it cannot provide sentinel information as to how formaldehyde interacts with cellular macromolecules. These effects on protein function may partially explain the synergistic effect formaldehyde has on genetic damage in mammalian cells [71,72]. Moreover, such effects on proteins may explain the association between NPC and consumption of salted fish containing nitrosamines like *N*-nitrosodimethylamine, as the cytochrome P450-mediated oxidation of these compounds can generate formaldehyde (Figure 1c) [5,7,73-76].

## Conclusion

NPC is a multifactorial disease thought to involve host, viral, and environmental factors. Evidence for an association between NPC and occupational exposure to formaldehyde remains debated, despite its classification as a human carcinogen [1-4,16-18]. Data described herein indicate that NO, acting through cGMP-independent pathways, can influence the EBV life cycle. As such, excessive environmental exposure to formaldehyde might perturb nitrosothiol homeostasis and potentiate EBV reactivation - thereby increasing the chance of basal epithelial cell infection. Data also suggest that protein stability and degradation are significantly affected by formaldehyde exposure; such changes might arise from direct formaldehyde modification of proteins, redox changes, or altered protein nitrosylation. Thus, concomitant with multiple perturbations caused by EBV latency programs, formaldehyde might additionally act additively or synergistically with EBV to promote epithelial cell transformation. Future investigations on these mechanisms may shed additional light as to the plausibility of epidemiological associations between formaldehyde exposure and NPC. Finally, the mechanisms presented herein might also be operative for other diseases associated with formaldehyde exposure that have etiologies involving EBV, such as lymphoproliferative disorders.

## List of abbreviations

ADH3: class III alcohol dehydrogenase; CYP2E1: cytochrome P450 2E1; EBNA: Epstein-Barr nuclear antigens; EBV: Epstein-Barr virus; FA: formaldehyde; FSH: *S*-formylglutathione; GSH: glutathione; GSNO: S-nitrosoglutathione; GSONH_2_: glutathione sulfinamide; GSSG: oxidized glutathione; HMGSH: *S*-hydroxymethylglutathione; IARC: International Agency for Research on Cancer; L-arg: L-arginine; LMP: latent membrane protein; MD^+^: methyldiazonium ion; NAD: Nicotinamide adenine dinucleotide; NF-κB: nuclear factor kappa-light-chain-enhancer of activated B cells; NDMA: *N-*nitrosodimethylamine; NO: nitric oxide; NOS: nitric oxide synthase; NPC: nasopharyngeal carcinoma; TNF: tumor necrosis factor; TPA: 12-*O*-tetradecanoylphorbol-13 acetate

## Competing interests

The authors declare that they have no competing interests.

## Authors' contributions

CT drafted the manuscript and constructed Figure 1. RG collaborated on all analytical and editorial decisions. Both authors read and approved the final manuscript.
